# Optimizing the Entrainment Geometry of a Dry Powder Inhaler: Methodology and Preliminary Results

**DOI:** 10.1007/s11095-016-1992-3

**Published:** 2016-07-11

**Authors:** Thomas Kopsch, Darragh Murnane, Digby Symons

**Affiliations:** 1 Department of Engineering, University of Cambridge, Trumpington Street, Cambridge, CB2 1PZ UK; 2 Department of Pharmacy, Pharmacology and Postgraduate Medicine, University of Hertfordshire, College Lane, Hatfield, AL10 9AB UK

**Keywords:** boundary-condition, cost-function, DPI, entrainment, optimization

## Abstract

**Purpose:**

For passive dry powder inhalers (DPIs) entrainment and emission of the aerosolized drug dose depends strongly on device geometry and the patient’s inhalation manoeuvre. We propose a computational method for optimizing the entrainment part of a DPI. The approach assumes that the pulmonary delivery location of aerosol can be determined by the timing of dose emission into the tidal airstream.

**Methods:**

An optimization algorithm was used to iteratively perform computational fluid dynamic (CFD) simulations of the drug emission of a DPI. The algorithm seeks to improve performance by changing the device geometry. Objectives were to achieve drug emission that was: A) independent of inhalation manoeuvre; B) similar to a target profile. The simulations used complete inhalation flow-rate profiles generated dependent on the device resistance. The CFD solver was OpenFOAM with drug/air flow simulated by the Eulerian-Eulerian method.

**Results:**

To demonstrate the method, a 2D geometry was optimized for inhalation independence (comparing two breath profiles) and an early-bolus delivery. Entrainment was both shear-driven and gas-assisted. Optimization for a delay in the bolus delivery was not possible with the chosen geometry.

**Conclusions:**

Computational optimization of a DPI geometry for most similar drug delivery has been accomplished for an example entrainment geometry.

## Introduction

### Establishment of Design Objectives for Inhaled Drug Delivery

Dry powder inhaler (DPI) therapy has increased in importance as a therapeutic modality for both pulmonary diseases such as asthma, and systemic diseases such as diabetes. In particular, the adoption of the Montreal Protocol banning the use of chlorofluorocarbon propellants accelerated development of DPIs as an alternative to the difficult reformulation of pressurized metered dose inhalers (1,2). In recent years, the suitability of DPIs for aerosolization and delivery of high drug payloads has been exploited, including the introduction of inhaled insulin and antibiotic products (3,4). There has also been an interest in developing bioequivalent (generic) DPI products as more established products reach the end of patent exclusivity. There is therefore a need to design and optimize DPI devices to achieve a variety of therapeutic outcomes in a diverse patient population ranging from those with healthy respiratory function to those with compromised respiratory function.

When designing a DPI, it is important to understand how particular design elements influence the release (emission) of aerosolized drug and consequently the device performance. It is therefore necessary to predict drug release profiles for particular entrainment geometries and to relate these profiles to the pharmaceutical objectives of drug delivery (two examples include: achieving bioequivalence, or maximizing peripheral airway deposition for systemic drug delivery). When predicting drug release profiles the entrainment mechanisms must be understood. In general, authors distinguish between gas-assisted, shear-force driven, capillary and mechanical fluidization (5). The fluidization mechanisms for different devices may be found in literature (*e.g*. (6,7)).

When designing an inhaler, several objectives have to be considered: First, drug should deposit in similar pulmonary airways, independent of the patient’s pulmonary function and ability to use the device (objective A). For example, van den Berge (8) stresses the importance of targeting the small airways for effective treatment of asthma and chronic obstructive pulmonary disease (COPD). Second, the drug release profile should be similar to a (pre-defined) desired released profile, for example an early-bolus release (objective B) (9). Additional objectives are to achieve de-agglomeration of drug powder (10,11) and low-manufacturing costs but these are beyond the scope of this paper.

It is accepted that the particle size distribution of the emitted aerosol is a significant factor in determining the site of deposition in the pulmonary airways (12), and the degree of de-agglomeration during device actuation is crucial in determining the particle size. However, the emission-time profile of that aerosol (even one with a size distribution suitable for targeting delivery to specific airways) is an area of DPI and inhaler design that remains under-explored. With the Adaptive Aerosol Delivery System (13), peripheral airway deposition of aerosol produced using a vibrating mesh nebulizer can be enhanced by limiting emission and inhalation of aerosol to 50 – 80% of the inspiratory cycle. A number of authors argue that delivering drug to a particular location of the lung correlates well to the pharmacokinetic profile (14). It has been shown that the timing of release correlates with the deposition location for nebulizers. Scheuch *et al*. (15) found that the site of drug deposition may be influenced by the timing of the bolus delivery. Heyder *et al*. (16) investigated in which ‘volumetric lung depth’ a bolus of drug deposits. Drug was introduced into the inhaled airflow after a particular fraction of the total volume was inhaled. Heyder *et al*. (16) showed experimentally that particle deposition was higher when the aerosol bolus penetrated deep into the lung. Similarly, Brand *et al*. (12) determined in which ‘lung volume element’ drug is likely to deposit by determining the probability of a particle depositing in the lung as a function of volumetric lung depth for an aerosol with a constant drug concentration. In the case of a bolus delivery the instant at which the drug was introduced into the airstream correlated (16) with the region to which drug was deposited.

Consequently, in this study it is assumed that the lung location to which drug will be delivered correlates well with the point in the inhaled tidal volume into which drug is introduced during inhalation. Although not disregarding the importance of the de-agglomeration processes, the argument is valid in cases where the DPI designer cannot easily influence the size of drug particles, if we assume a powder that has been optimized for its ease of dispersion (17,18). Following this argument, it is important to optimize DPIs to release drug at the correct instant with respect to the fraction of inhaled volume (*x*) rather than with respect to the inhalation time (*t*).

### Computational Fluid Dynamics for DPI Design

Computational fluid dynamic (CFD) analysis refers to the use of computational techniques to solve fluid flow problems. The application of CFD techniques in inhaled drug delivery has increased in recent years for prediction of pulmonary aerosol deposition, but also to improve understanding of aerosolization processes, *e.g*. to predict the performance of DPIs (19). CFD has also been used to assess device resistance (20,21), track the path of individual particles (21) and to investigate particle de-agglomeration in DPIs (10,22,23).

In CFD analysis for DPIs the particulate (drug) phase can either be ignored (a single phase approach considering air flow only) or modelled using a multiphase approach. The majority of reported studies using multiphase approaches for simulation of DPIs have used Eulerian–Lagrangian (EL, particle-tracking) approaches (24). EL approaches make it difficult to study the fluidization process of a powder bed due to the large number of particle-particle interactions. It is for this reason that we have recently introduced Eulerian-Eulerian (EE) approaches (9,25) for investigation of DPIs.

**Fig. 1:**
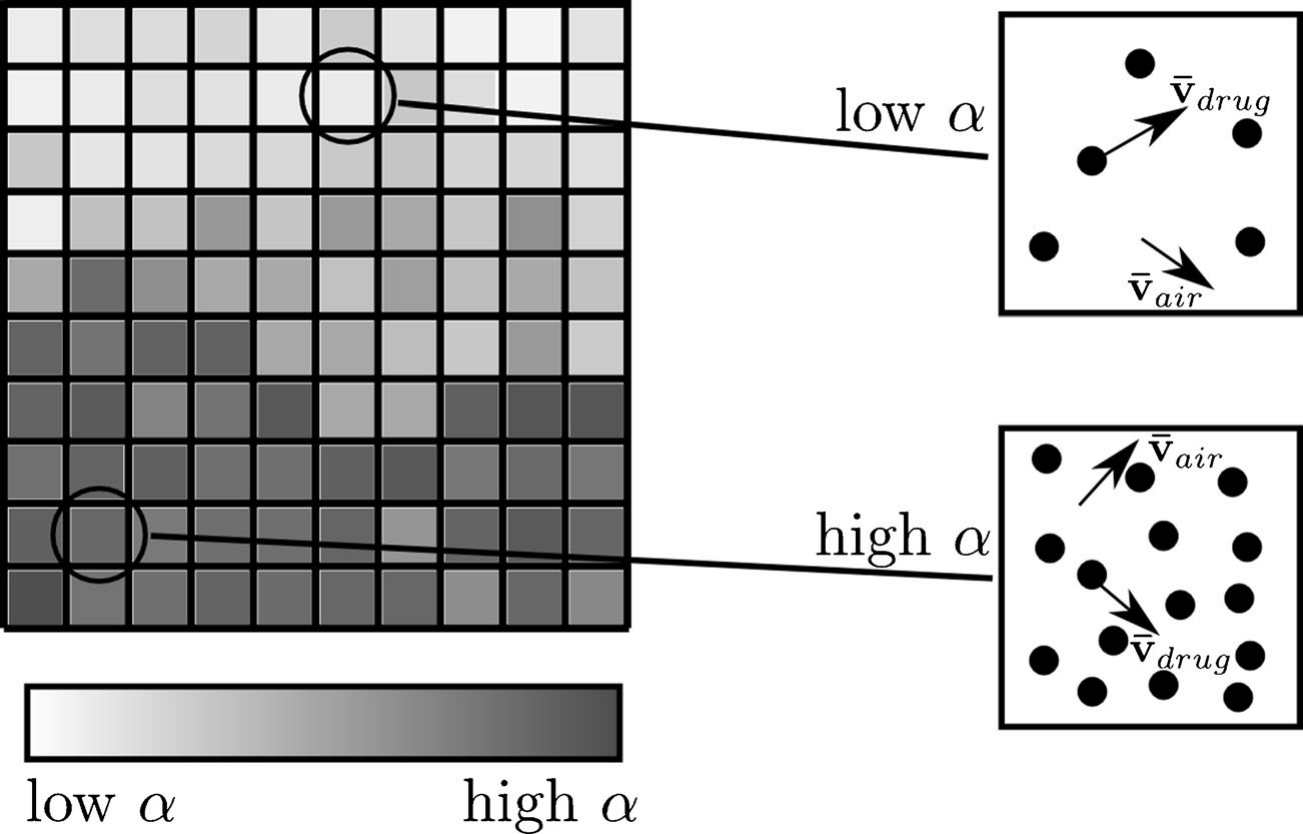
Idealized representation of the EE approach. The volume fraction *α* indicates the density of particles.

In the EE approach, the drug particles are modelled as a second continuous phase and the interaction between the gas and the granular phase is modelled. A new variable, the volume fraction (*α*) is introduced to keep track of the local fraction of drug phase. EE approaches are often used in the study of other fluidization processes (26,27). As in other CFD approaches the domain of interest is split into a large number of cells. For each cell the Eulerian-Eulerian solver solves for a number of variables including *α*, the average velocity of drug particles ${\overline{\mathbf{v}}}_{drug}$ and the average velocity of the gaseous phase ${\overline{\mathbf{v}}}_{air}$. As shown in Fig. 1 the value of *α* indicates the number of particles in that cell. In the EE approach, all phases, including the particulate phases, are modelled as continuous fluids and consequently mass, momentum and energy conservation laws are applied to all phases. In many cases, these equations look similar to the standard Navier–Stokes equations. The kinetic theory for granular flow (KTGF) (28) is one method to model the constitutive behaviour of a granular phase in the EE approach. KTGF makes predictions about stresses and phase interaction terms in the governing equations of the fluid.

For modelling purposes, the applied boundary condition at the outlet of the inhaler is usually a constant flow rate or a transient pressure boundary condition (25). A constant flow rate boundary condition is prescribed in regulatory inhaler testing (29,30). However, physiologically relevant inhalation flow-rate profiles are more complex. As shown by de Koning *et al*. (31,32) the peak inhalation flow (PIF), total inhalation time and other inhalation profile variables depend on the device resistance and the type of disease. It is of interest to develop a DPI modelling approach that can incorporate the complexity of inter-individual variability in inhalation performance and flow-rate profiles. For this reason the experimental data from de Koning *et al*. (31) was employed to model resistance-dependent inhalation profiles and to establish the outlet boundary conditions for a CFD simulation. In particular it is of interest to simulate the drug release profiles during the simulated inhalations in order to guide the design and optimization of different DPI geometries.

There has been relatively little prior work on the use of computational optimization methods for the design of DPIs. The aim of this study was to address the knowledge gap in the application of computational fluid dynamics (CFD) in the numerical optimization of DPIs. In order to achieve the aim, optimization cost functions have been developed for the quantitative assessment of the performance of device entrainment geometries for a range of simulated inhalation profiles. As an example of the methodology, these cost functions have been applied in an optimization framework in order to improve the drug emission performance of a simple entrainment geometry.

## Methods

### Cost Function Development

The goal of numerical optimization is to find an argument **x** that minimizes a cost function *C*(**x**), where **x** consists of a number of design variables. In engineering applications the cost *C* measures some quality of a design. For example, *C* could measure the manufacturing cost or energy consumption. In this study we only consider drug delivery performance of a DPI device. We propose two cost functions that measure entrainment and emission performance. The optimization goal is to minimize these functions. Cost function A prefers devices that can emit drug to similar pulmonary airways (12) (*i.e*. volumetric lung depth), independent of the patient’s respiratory function. Cost function B prefers devices that achieve a desired drug release profile (*e.g*. early bolus). The cost functions are developments of earlier concepts presented by the authors of this study (9,25).

For the first design objective, the goal is to achieve the ‘most similar drug delivery’, *i.e*. the drug should penetrate to the same pulmonary region for different patients. To test this objective a large number of patients with different inhalation manoeuvres could be considered. However, to illustrate the approach only two inhalation profiles, 1 and 2, are considered in this study.

**Fig. 2:**
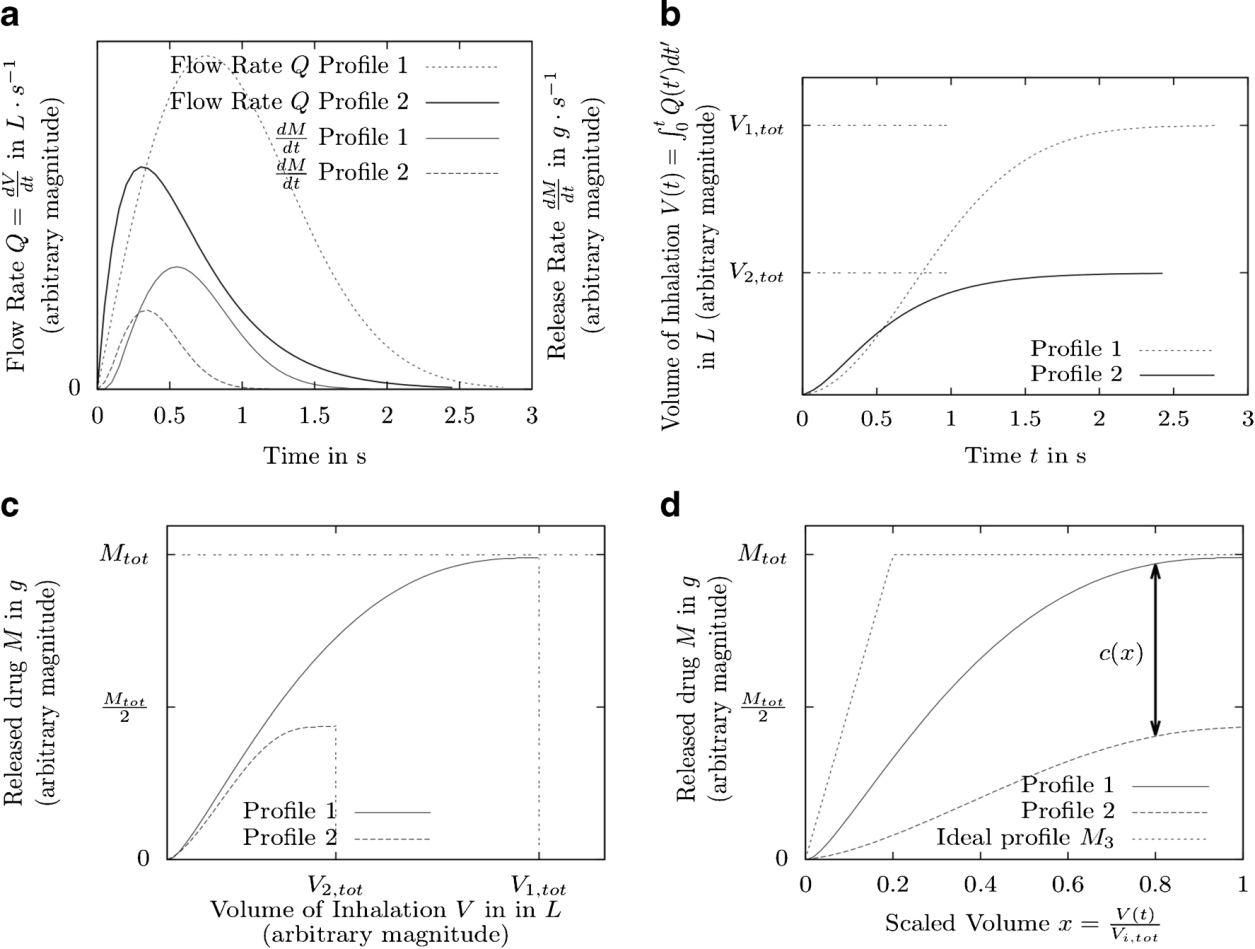
Idealized representations of inhalation flow rate (*Q*), inhaled volume ($V(t)$), released drug (*M*) and scaled volume (*x*) as functions of time or each other. Sample data for clarification purposes only. This Fig. is similar to Fig. 1 in (1). (**a**) Flow rate and release rate *vs*. time. (**b**) Volume of inhalation *vs*. time. (**c**) Released drug *vs*. volume. (**d**) Released drug *vs*. scaled volume.

In general, patients have different inhalation profiles; *i.e*. when they inhale through the device, they produce different volumetric flow rates *Q*_1_ (*t*) and *Q*_2_ (*t*) (see example profiles in Fig. 2a) leading to different drug emission rates (*dM*/*dt*) (Fig. 2a). The integral (Eq. 1) gives the inhaled volume as a function of time, *t*:

1 $$ {V}_i(t)={\displaystyle {\int}_0^t{Q}_i\left({t}^{\prime}\right)dt^{\prime }} $$

where *i* = patient 1 or 2. The total inhaled volume (see Fig. 2b) is given by Eq. 2:

2 $$ {V}_{i,\  Tot}={\displaystyle {\int}_0^{\infty }{Q}_i\left({t}^{\prime}\right)dt^{\prime }} $$

After carrying out a CFD simulation of entrainment, the emitted dose profile *M*_*i*_ can be calculated. *M*_*i*_ is the mass of drug that has left the device at one point and can be expressed as either a function of time *M*_*i*_ (*t*) or inhaled volume *M*_*i*_ (*V*) (see Fig. 2c). *M*_*Tot*_ is the total drug amount in the primed dosing unit (*i.e*. capsule, blister, metering cup). *M*_*i*_ (*V*) may be scaled along the *V*-axis by defining the scaled volume (Eq. 3):

3 $$ \begin{array}{ccc}\kern1em x=\frac{V_i}{V_{i,T\kern-.12em ot}}\kern1em & \kern.5em \mathrm{where}\kern1em & \kern.5em 0\le x\le 1\kern1em \end{array} $$

This gives *M*_*i*_(*V*) = *M*_*i*_(*x V*_*i*,*Tot*_), (see Fig. 2d).

The device will ideally have released the same amount of drug for patients 1 and 2 when the same fraction *x* of the total inhaled air *V*_*i*,*Tot*_ has been inhaled. It follows that identical dose emission would be represented by Eq. 4, for all *x*.

4 $$ {M}_1\left(x\ {V}_{1, Tot}\right)={M}_2\left(x\ {V}_{2, Tot}\right) $$

Since it is impossible to achieve Eq. 4 exactly, the design objective is, therefore, to minimize the difference between the drug release profiles (Eq. 5) for all *x*:

5 $$ c(x) = \left|{M}_1\left(x\ {V}_{1, Tot}\right) - {M}_2\left(x\ {V}_{2, Tot}\right)\right| $$

Figure 2d shows the same release profiles as in Fig. 2c, but as a function of scaled volume *x*. It is hoped to minimize *c*(*x*) = 0 for all *x*. A possible cost-function to achieve this is therefore Eq. 6:

6 $$ {C}_A={\displaystyle {\int}_0^1c(x)dx={\displaystyle {\int}_0^1\left|{M}_1\left(x{V}_{1, Tot}\right)-{M}_2\left(x{V}_{2, Tot}\right)\right|dx}} $$

Minimizing *C*_*A*_ will ensure ‘most similar drug delivery’ for different patients. Note that since CFD solves discretized versions of the governing equations in practice the integral (Eq. 6) has to be approximated as a summation.

Minimizing the cost function *C*_*A*_ guarantees a device airflow geometry that can achieve the highest similarity of the emission-volume profile. However, it cannot be guaranteed that this emission profile is also a desired one. It depends on the desired therapeutic application whether, for example, either an early bolus delivery (to reach peripheral airways) or continuous emission (to achieve deposition throughout all airways) is required. We should, therefore, also consider comparing the release profile with a desired, idealized release profile. As an illustration, if for a particular medical application an early bolus delivery of drug is desired, an idealised early bolus profile (see Fig. 2d) can be defined using Eq. 7:

7 $$ {M}_3(x)=\left\{\begin{array}{c}{M}_{tot}\frac{x}{x_0}\kern0.5em \mathrm{if}\kern0.5em x<{x}_0\kern.5em \\ {}{M}_{tot\kern0.5em }\mathrm{if}\kern0.5em x\ge {x}_0\end{array}\kern1em \right\} $$

This is a ‘ramp’ function, *i.e. M*_3_ (*x*) represents an early-bolus profile, which increases linearly from 0 to *x*_0_ and is constant from *x*_0_ to 1. This is more realistic compared to (9), where an ideal step function was used. To evaluate how closely the emitted dose profile from our device matches the idealised profile the cost function given by Eq. 8 has been used:

8 $$ {C}_B={\displaystyle {\int}_0^1c(x)dx=}{\displaystyle {\int}_0^1\left|{M}_1\left(x{V}_{1, Tot}\right)-{M}_3(x)\right|dx} $$

Minimizing cost function *C*_*B*_ gives a geometry with the desired release behaviour. To also achieve ‘most similar drug delivery’ between different patients requires simultaneous minimization of cost function *C*_*A*_.

### Boundary Conditions

When conducting a CFD analysis, boundary conditions (BCs) have to be specified. At the inlet a (constant) atmospheric pressure is usually specified. The applied boundary condition at the outlet should resemble the flow-field generated by a real patient inhaling through the device.

In many experimental and computational studies a constant flow rate *Q* or a constant pressure drop ∆*p* is applied (10,21,33). A constant flow rate boundary condition simplifies experimental studies and regulatory testing (29,30). However, if much of the drug entrainment occurs before the PIF is reached, or if the PIF is not sustained for very long, then using a constant flow rate BC may not model the real entrainment behaviour accurately. Zimarev *et al*. (25) went some way to addressing this issue by applying a pressure ramp BC of constant $\frac{dp}{dt}$ for a fixed simulation time to represent the start of an inhalation. This is a good improvement if entrainment occurs early in the inhalation manoeuvre. However, some devices have not released all the drug dose before PIF is reached (34). A fixed simulation time is therefore only acceptable if most of the drug leaves the device within the simulated time. A possible solution to these problems is to specify the flow rate *Q* (*t*) as a function of time at the outlet and model a whole inhalation profile (or at least the first part of the profile until drug emission is complete).

**Fig. 3:**
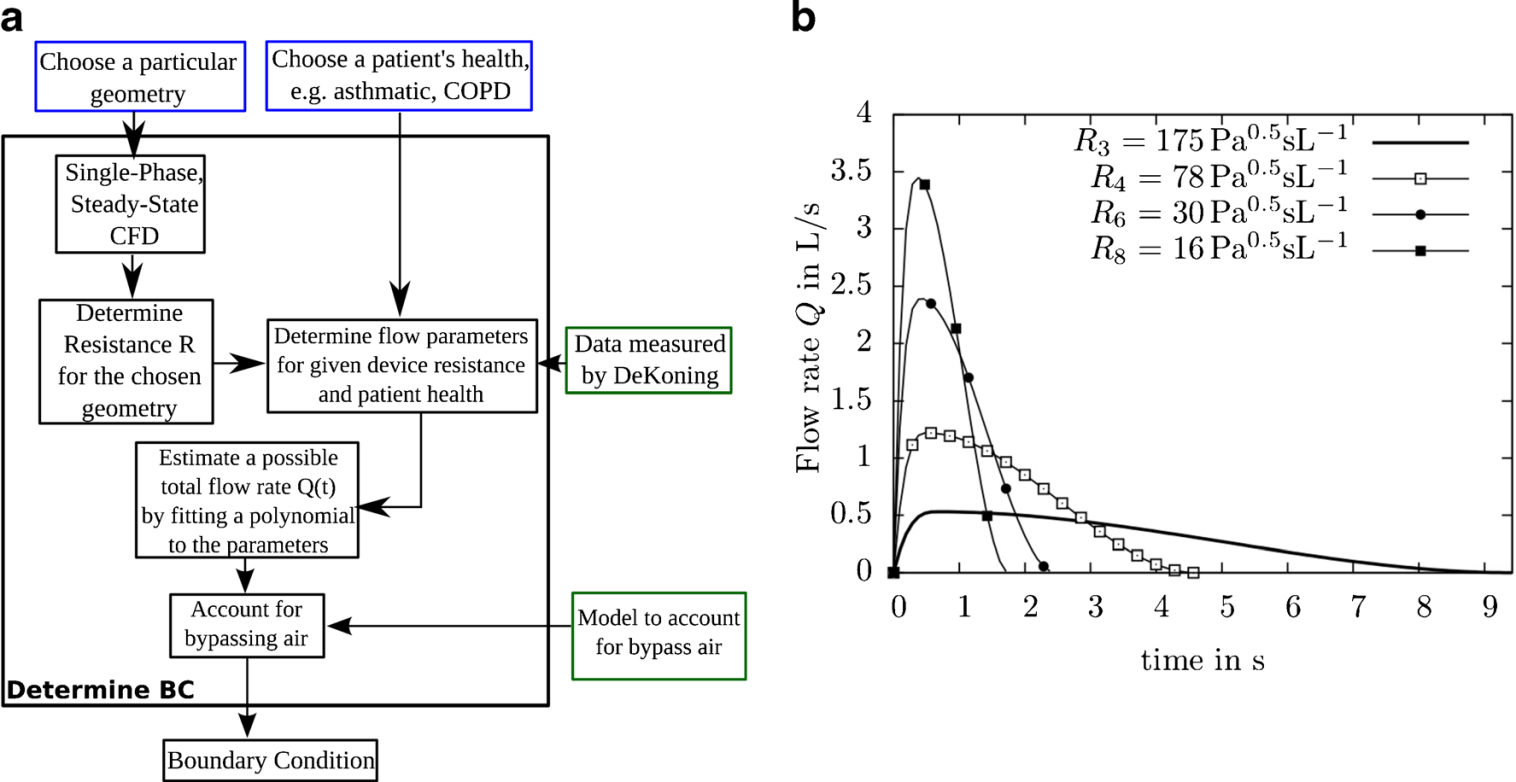
Determining the boundary condition and examples of release profiles for different resistance *R*. (**a**) Determine transient boundary conditions. (**b**) Modelled *Q* (*t*) function for different resistances *R*_3_ - *R*_8_. Piecewise defined polynomials were fitted to data from de Koning *et al*. (31,32).

The applied *Q* (*t*) BC should be as realistic as possible. In practice measured flow rate profiles vary significantly as a function of device resistance *R*, *i.e*. a lower flow rate is expected to be produced by any given patient for high resistance devices and *vice versa*. For example, de Koning *et al*. (31,32) plotted the flow rate as a function of time for two different device resistances and health of patient. Several important (averaged) parameters of the inhalation profile were reported, such as PIF, the time when the PIF occurs *t*_*PIF*_, and total time of inhalation *t*_*tot*_ as a function of resistance and patient health. Thus, it is possible to fit a polynomial to these points. The following procedure was applied (see Fig. 3a):

1. Determine the resistance *R* in $QR = \sqrt{\varDelta p}$ by running a steady-state CFD simulation (using simpleFOAM from OpenFOAM (35)) where a constant pressure difference ∆*p* is applied. *Q* is found from the simulation. Hence, $R=\sqrt{\varDelta p}/Q$ is calculated.
2. Interpolate the data from de Koning *et al*. (31,32) to find relevant flow field parameters (*PIF*, *t*_*PIF*_, *t*_*tot*_) for the particular value of *R* and patients’ disease state.
3. Model a flow rate function Q (*t*) from these parameters.
4. Account for a fraction of bypassing air: Many DPI devices allow the majority of inhaled air flow to bypass the drug entrainment geometry, *e.g*. (21).

In principle, a wide range of functions could be fitted to the parameters in step 3 to model a flow rate function *Q* (*t*). In this case, however, *Q* (*t*) was modelled using two piecewise-defined 3rd order polynomials f (*t*) and *g* (*t*):

9 $$ Q(t)=\left\{\begin{array}{c}f(t)\kern0.75em \mathrm{if}\kern0.75em t<{t}_{PIF}\\ {}g(t)\kern0.75em \mathrm{if}\kern0.98em t\ge {t}_{PIF}\\ {}0\kern2.25em \mathrm{if}\kern0.75em t\ge {t}_{tot}\end{array}\right\} $$

The first polynomial, *f* (*t*), links the onset of the inhalation with the PIF. The second polynomial connects the PIF with the end of the inhalation. The coefficients of the polynomials f (*t*) and g (*t*) can be determined numerically by imposing a number of conditions at the start and end of the inhalation:

1. At *t* = 0: *Q*(0) = *f*(0) =0
2. At *t* = *t*_*tot*_: *i.e. Q* (*t*_*tot*_) = *g* (*t*_*tot*_) =0
3. *Q* (t) drops slowly towards the end of the inhalation, *i.e. Q* ′ (*t*_*tot*_) = 0.

At the transition of the two polynomials, *i.e*. at point (*t*_*PIF*_, *PIF*), it is required that

4. *Q* (*t*) is continuous, *i.e. f* (*t*_*PIF*_) = g (*t*_*PIF*_) = PIF
5. *Q* (*t*_*PIF*_) is a maximum, *i.e. f*′ (*t*_*PIF*_) = *g*′ (*t*_*PIF*_) = 0.
6. It was also assumed that the second derivative is continuous, *i.e. f*″ (*t*_*PIF*_) =g″ (*t*_*PIF*_).

Using these conditions, the coefficients of the polynomials were determined. Fig. 3b shows example fits to data from de Koning *et al*. (31,32) for different device resistances.

In this study the method described above, using de Koning’s data (31,32), was used to generate the idealized inhalation profile *Q*_1_ (*t*) for an average patient for each device geometry considered. The second inhalation profile *Q*_2_ (*t*) was generated in a similar way, but with a lower PIF. The PIF of ${Q}_2(t)$ was chosen to be one standard deviation below that of *Q*_1_ (*t*). The time to PIF ${t}_{PIF}$ and the duration ${t}_{tot}$ were the same for both ${Q}_1(t)$ and ${Q}_2(t)$ (in contrast to the example profiles shown in Fig. 2).

### CFD and Optimization

#### CFD Methodology

As an example implementation, the cost functions (section 2.1) and the dynamically generated boundary conditions (section 2.2) were applied in a CFD optimization routine. First, a simple 2D geometry was parametrized in terms of five design variables: $a,\;b,\;c,\;d,\;e$. Using a Python-script (36) the geometry was generated depending on these design parameters. BlockMesh (OpenFOAM (35)) was used to mesh the geometry. The Eulerian-Eulerian solver twoPhaseEulerFoam (OpenFOAM) was used to simulate the drug release through the device for high and low flow-rate boundary conditions. Transient boundary conditions were applied using the OpenFOAM library swak4Foam (37). A Python-script (36) determined the drug-release profiles as a function of the scaled inhaled volume x for each boundary condition and then calculated the cost functions ${C}_A$ and ${C}_B$.

A total cost function *C*_*tot*_ = *w*_*A*_*C*_*A*_ + *w*_*B*_*C*_*B*_ was calculated (where ${w}_A$ and ${w}_B$ are weightings). The cost function ${C}_{tot}$ was minimized by systematically changing the geometry using a steepest gradient-descent method, see *e.g*. (38).

#### Definition of Design Space for Optimization and CFD Studies

**Fig. 4:**
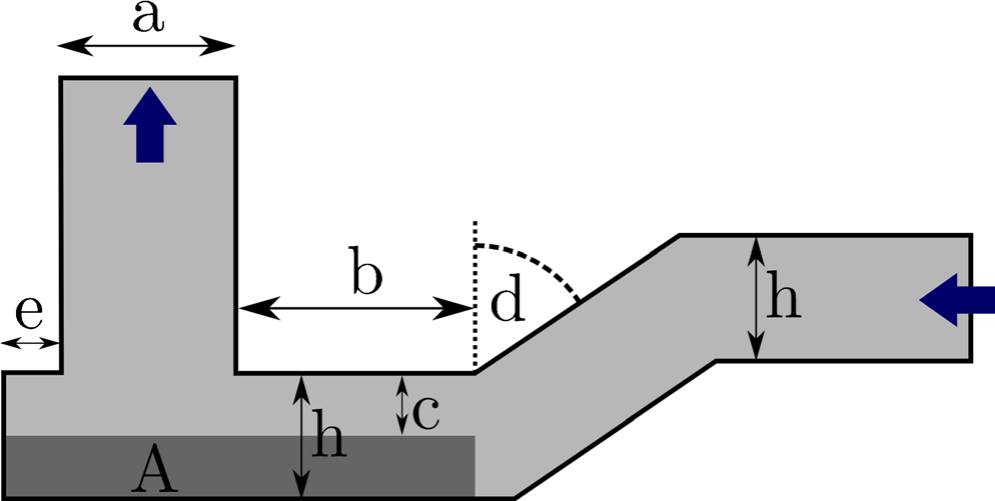
Parameterized entrainment geometry.

A simple DPI device was considered in this study (Fig. 4). The geometry was chosen so that it could represent a range of types of design of existing DPI. The device consists of an inlet, an entrainment area and an outlet. A fixed volume of drug of 3 mm^2^ was initially placed into the entrainment area (note that in 2D simulations, a volume may be represented by an area A). The size of the outlet is a, the height of the capsule/blister and the inlet is h. The capsule/blister may or may not be filled with drug completely. The separation between the drug surface and the upper boundary of the capsule is c. d is the angle between the inlet tube and the capsule. The amount of drug A filled into the device was constant. The height, h, of the capsule was therefore calculated from $h=c+A/\left(e+a+b\right)$.

Table I shows the lower and upper boundaries of the design variables. The values of these boundaries were chosen to give geometries of realistic sizes. Daniher and Zhu (5) identified different types of drug entrainment in DPIs, *i.e*. shear-driven and gas-assisted entrainment. This parameterized device is designed to allow different entrainment behaviour for different values of the design variables. For example, a gas-assisted entrainment may be expected when e and c are small and d is close to $90{}^{\circ}$. Such a geometry approximates the bathtub shaped blister found in some DPIs where the foil lid is pierced to from an inlet and outlet (39). On the other hand, shear-driven entrainment, *e.g*. (40), may be expected when e and c are large. In addition, the resistance may be influenced by varying any of the parameters, since the latter change the airflow paths within the entrainment region of the DPI device.

#### Eulerian-Eulerian Modelling

When the volume fraction of the drug phase is high, the drug may significantly influence the flow field. For this reason an Eulerian-Eulerian (EE) approach was used in this study to simulate drug release profiles. When the volume fraction of drug is very high, the EE approach is more computationally efficient than an Eulerian–Lagrangian (EL) particle tracking approach. The models and parameters used in this study are given in Table II. Lactose powder was simulated in this study in order to compare the computational results with experimental results from (41) for validation purposes. While the real lactose powder comprises of particles with a distribution of particle sizes, in this study a mono-dispersed powder was simulated. Lactose particles are usually used as diluent, also known as carrier particles (42,43) and have high average diameter compared to the drug, excluding the fines. In this study de-agglomeration was not simulated. Although a simplification for the purposes of the current study, it was assumed that the drug particles would de-agglomerate from their lactose carrier during or after entrainment. Delivery of active drug is therefore assumed to be proportional to the amount of entrained lactose powder.

#### Optimization Algorithm

In this study a steepest gradient descent optimization algorithm was used to minimize the total cost function *C*_*tot*_. Successful implementation of gradient descent relies on determining the search direction **p** which is the direction of steepest descent **p** = − ∇*C*_*tot*_ and on deciding a suitable step length α (38). To enhance the performance of the optimization algorithm, all five design variables were scaled to confine values between 0 and 1. For example, a^*^, the scaled version of design variable *a*, was calculated using Eq. 10.

10 $$ {a}^{*} = \frac{a-{a}_{low}}{a_{high}-{a}_{low}} $$

*a*_*low*_ and *a*_*high*_ are the lower and upper boundary of a, respectively. Similarly, all other design variables were scaled. This gives a vector of scaled design variables:

11 $$ \mathbf{x}=\left({a}^{*},{b}^{*},{c}^{*},{d}^{*},{e}^{*}\right) $$

If, at step *k* of an optimization process, the current set of design variables is **x**_***k***_ = (a*, b*, c*, d*, e*), a new choice of design variables, for the next step (*k* +1), may then be evaluated as follows (38):

12 $$ {\mathbf{x}}_{\boldsymbol{k}+1} = {\mathbf{x}}_{\boldsymbol{k}}+\alpha\ \mathbf{p} $$

In this study, the search direction **p** was determined by approximating − ∇*C*_*tot*_ using a finite difference method. Using a backtracking line search a suitable step length α was determined (38). Since this is a constrained optimization problem a penalty term was applied if the constraints were exceeded. In order to avoid convergence to a local minimum, the same optimization algorithm was repeated for different (random) initial values of the design variables.

## Results & Discussion

### Experimental Validation of the CFD Approach

**Fig. 5:**
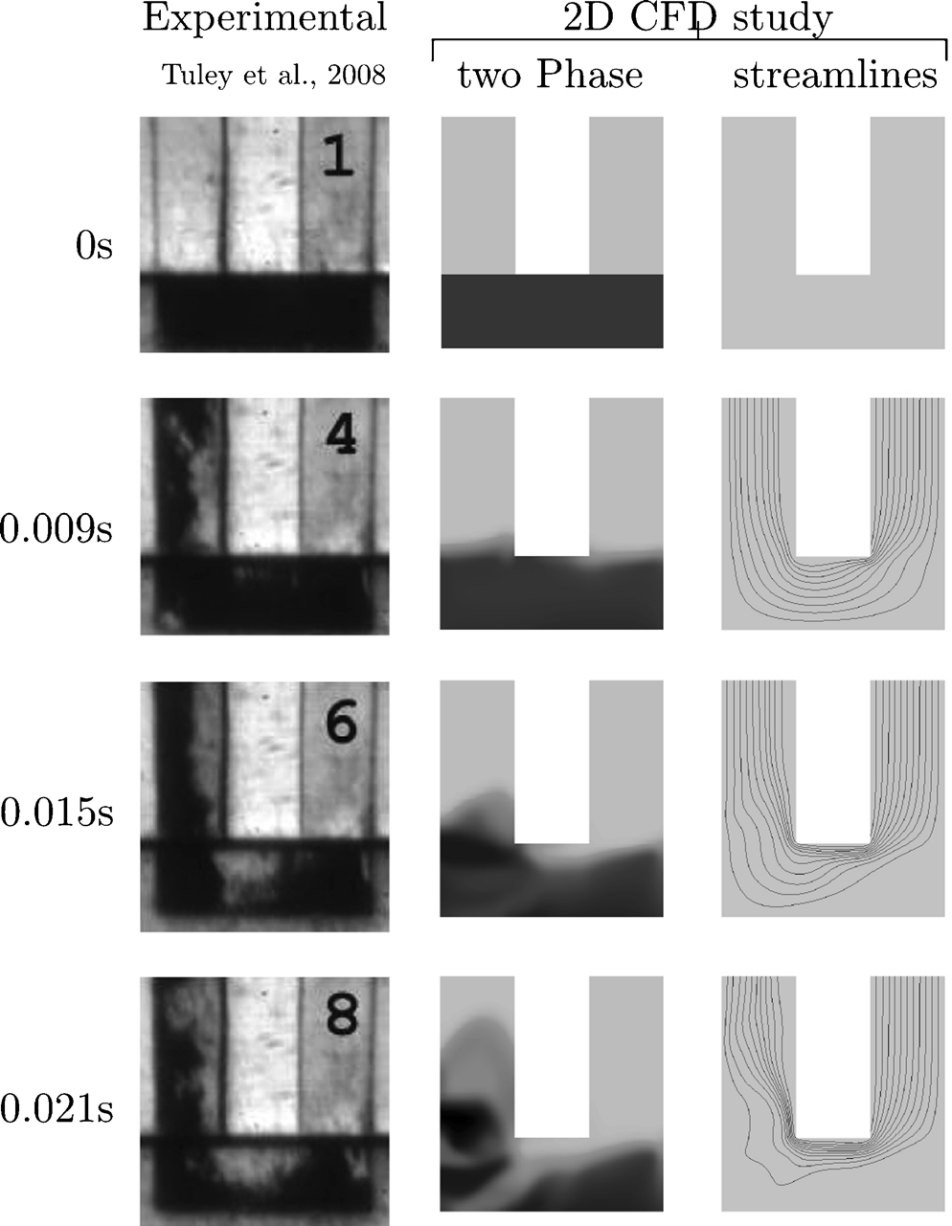
Comparison of experimental data (Tuley *et al*. (41), Fig. 6) with numerical simulation. The inlet is the right leg, the outlet is the left leg. Tuley *et al*. (41) filled lactose powder (16% fines) into the entrainment device.

The CFD method developed and reported in this study has been validated by comparison with entrainment experiments performed by Tuley *et al*. (41). In Tuley’s study, a constant pressure gradient $\frac{dp}{dt}$was applied at the outlet of three different entrainment geometries. Each geometry permitted entrainment by a different combination of shear and gas assisted mechanisms. The entrainment geometries were transparent and a video camera was used to record the entrainment of particles. In order to validate the CFD approach chosen in this study, one of these entrainment geometries was modelled using the CFD approach described in Sections 2.3.1 – 2.3.3. For the geometry selected (see Fig. 5) the initial entrainment mechanism is gas-assisted, but it transitions to shear driven as evacuation progresses.

Figure 5 shows images of an entrainment device for different times as lactose powder (16% fines) was entrained when a pressure gradient of $\frac{dp}{dt}$ = −30 kPa s^−1^ was applied at the outlet of the device (41). For comparison, the numerical results (EE CFD) are shown next to the experimental results. Tuley *et al*. (41) recorded the first frame at $t=0.033\mathrm{s}$, and therefore all CFD modelling timepoints are given relative to this time. The plots of streamlines shown in Fig. 5 have been compiled from the air velocity solution (EE CFD). The streamlines are curves that are tangent to the velocity vector of the air at any given time. Typically, streamlines converge when the speed of the fluid increases. Therefore, plots of streamlines may be used to analyse how the flow is affected by the presence of the drug powder: Initially, the volume fraction of drug α is 0.49, see Table II. Once drug is moved away from the powder bed surface, a thin channel of air is shaped. The streamlines converge towards each other in this channel. Thus, air flows preferably through this channel. The results from the simulation appear to be similar to the experimental results with the formulated powder being released over similar timescales and in a similar process in the model as in the experimental observations.

### mesh Independence Study

**Fig. 6:**
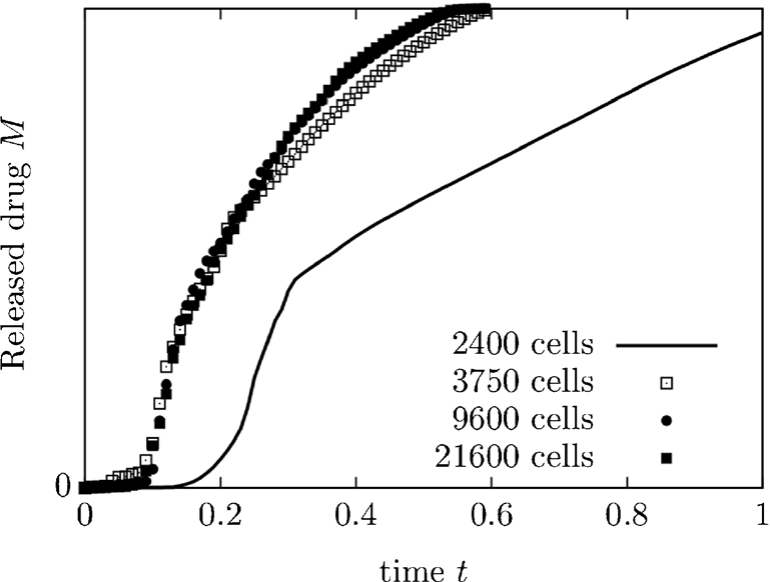
Mesh independence study.

The accuracy and the computational costs of CFD simulations are influenced by the type of chosen mesh (44) . In general, a high mesh density results in more detailed and accurate results, but also higher computation time. It is important to verify that the numerical results are independent of mesh resolution. Small changes in the mesh should not result in large changes in the results. A mesh-independence study may be conducted by plotting one variable for different meshes. Fig. 6 shows the result of a mesh-independence study: For a particular geometry $\left({a}^{*},{b}^{*},{c}^{*},{d}^{*},{e}^{*}\right)=\left(0.5,\;0.5,\;0.5,\;0.5,\;0.5\right)$, the number of cells used to mesh the geometry was varied. It appears that meshes with more than 3750 cells are sufficient to achieve mesh independence.

### Exploration of Design Space for DPI Optimization

Before the design space could be explored two parameters had to be set. First, the relative weightings ${w}_A$ and ${w}_B$ of the cost functions *C*_*A*_ and *C*_*B*_ had to be set. Second, the value of ${x}_0$ in the ideal drug release profile *M*_3_ (*x*) had to be set (see Eq. 7). Initially, the weightings were set to **w** = (*w*_*A*_, *w*_*B*_) = (1, 1), *i.e*. both cost functions were weighted equally. However, *C*_*A*_ tended to be (much) smaller than *C*_*B*_. This implies that for the modelled (pierced blister) geometry, objective A (similar drug release for different patients) is comparatively easier to achieve than objective B (similarity to a desired drug release profile). Consequently, the weighting was changed to **w** = (40, 1) in a second optimization process. These weighting values imply that most emphasis is put on achieving objective A. In this case objective B is (almost) ignored.

**Fig. 7:**
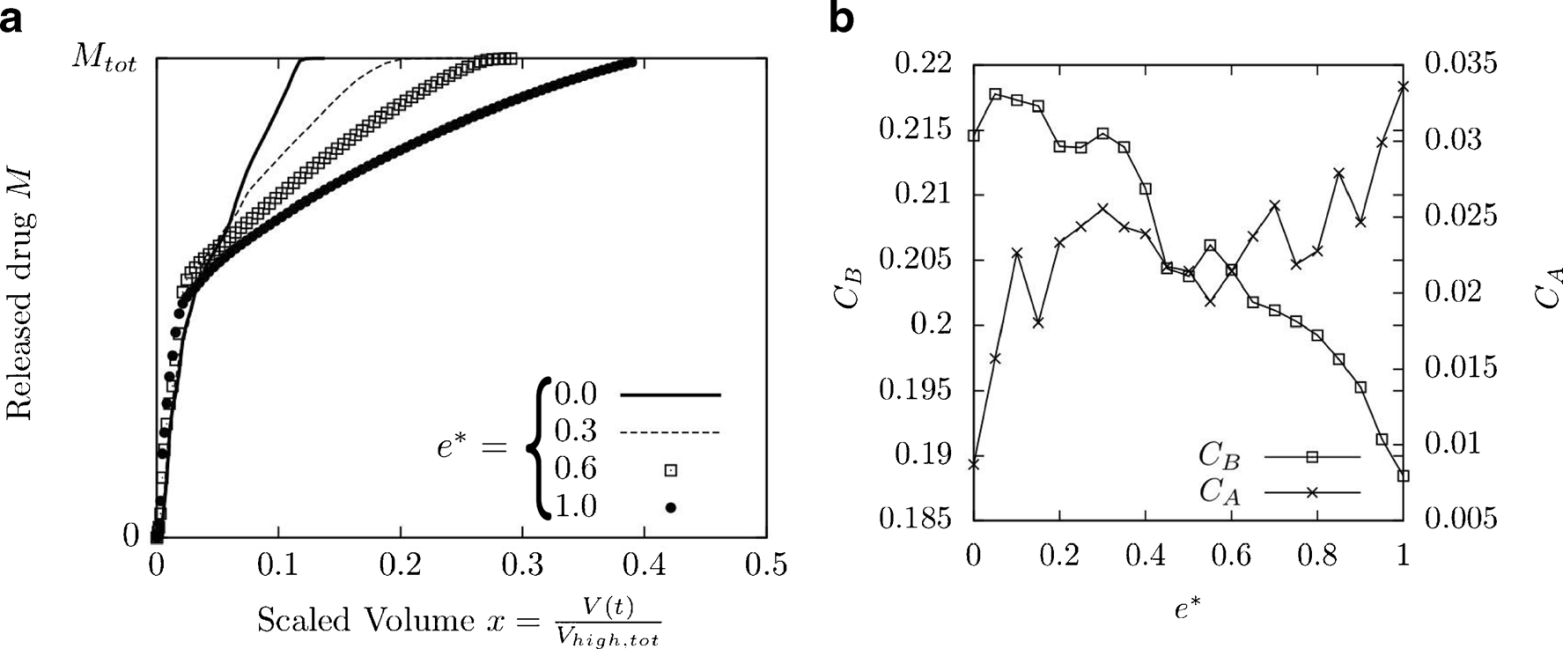
Release profile and costs for different values of the length of the compartment end e*. All other parameters were kept constant: (a*, b*, c*, d*) = (0.76, 0.26, 0.20, 0.38). (**a**) Example release profiles for four different values of ${e}^{*}$. The inhalation profiles applied were those of a weaker patient (*Q*_2_(*t*)). (**b**) Value of the cost functions *C*_*A*_ and *C*_*B*_ as e* is varied.

In addition, two different values for *x*_0_, the duration of drug release in the ideal release function *M*_3_ (x), were examined (see Eq. 7). A value of *x*_0_ = 0.2 gives an ideal early bolus drug release, while a higher value of *x*_0_ = 0.5 gives a more continuous release profile. As an example, the results for the scaled release profile and the corresponding cost functions are presented in Fig. 7 when just a single parameter, the length of the compartment end *e*^∗^, was varied, while all other parameters were kept constant. Fig. 7a shows that early delivery of drug is achieved for small values of *e*^∗^, while a more continuous delivery is achieved for larger values of *e*^∗^. Fig. 7b indicates a trade-off between costs *C*_*A*_ and *C*_*B*_: A high value of *e*^∗^ results in a continuous drug delivery and hence a small value of *C*_*B*_ (for *x*_0_ = 0.5). However in this regime, of longer duration delivery, differences between the emission profiles for patients 1 and 2 become more significant and so ${C}_A$ increases.

The results show that powder entrainment can be influenced by changing design parameters. In particular, a better early bolus delivery may be achieved. This phenomenon is consistent with experimental findings. For example, Tuley *et al*. (41) compared different entrainment devices and found that both the fluidization process and the timescale of fluidization is influenced by the choice of geometry.

### Optimization Results

**Fig. 8:**
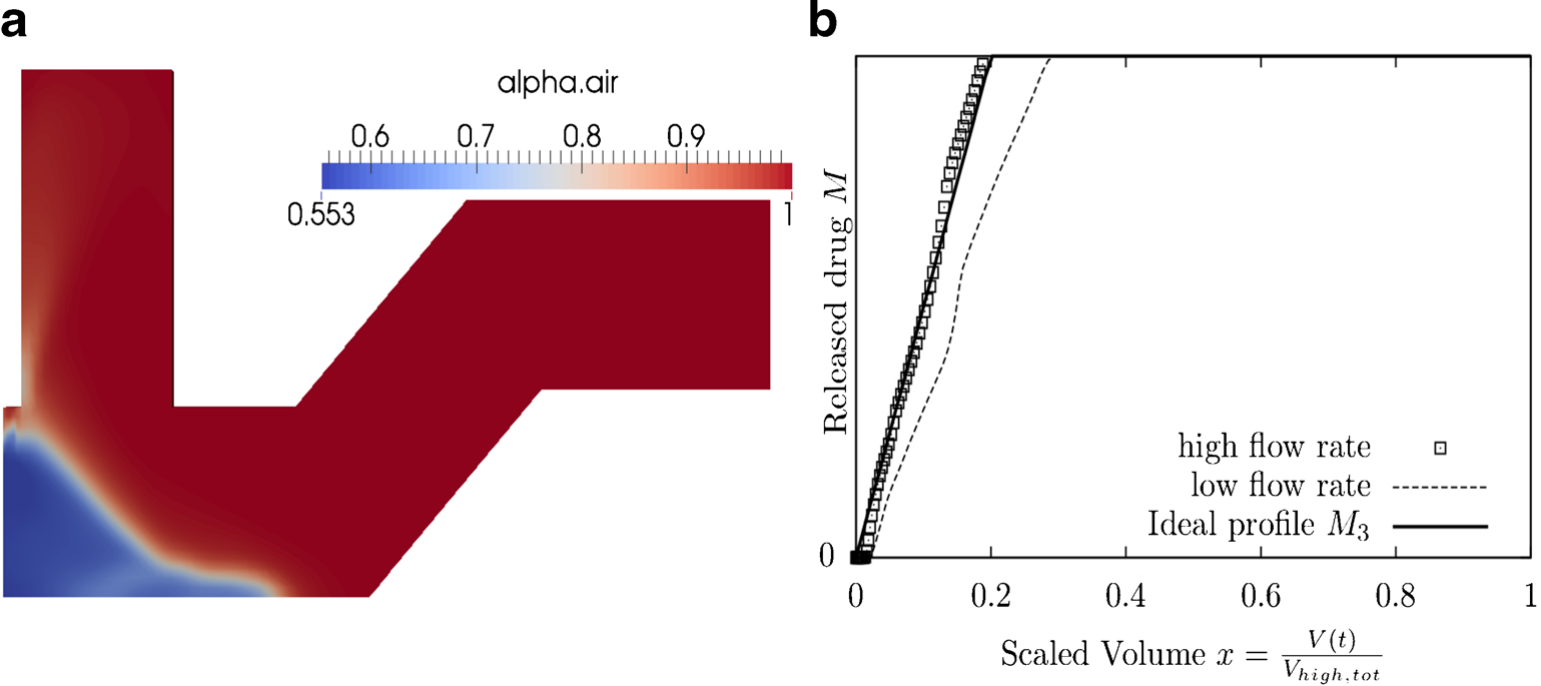
*C* = *C*_*A*_ + *C*_*B*_, *x*_0_ = 0.2, the optimal set of design variables is **x** = (0.68, 0.10, 0.64, 0.47, 0.08). (**a**) The optimal geometry and the volume fraction *α* at time *t* = 0.2s. (**b**) Scaled drug release profile for high and low flow rate.

**Fig. 9:**
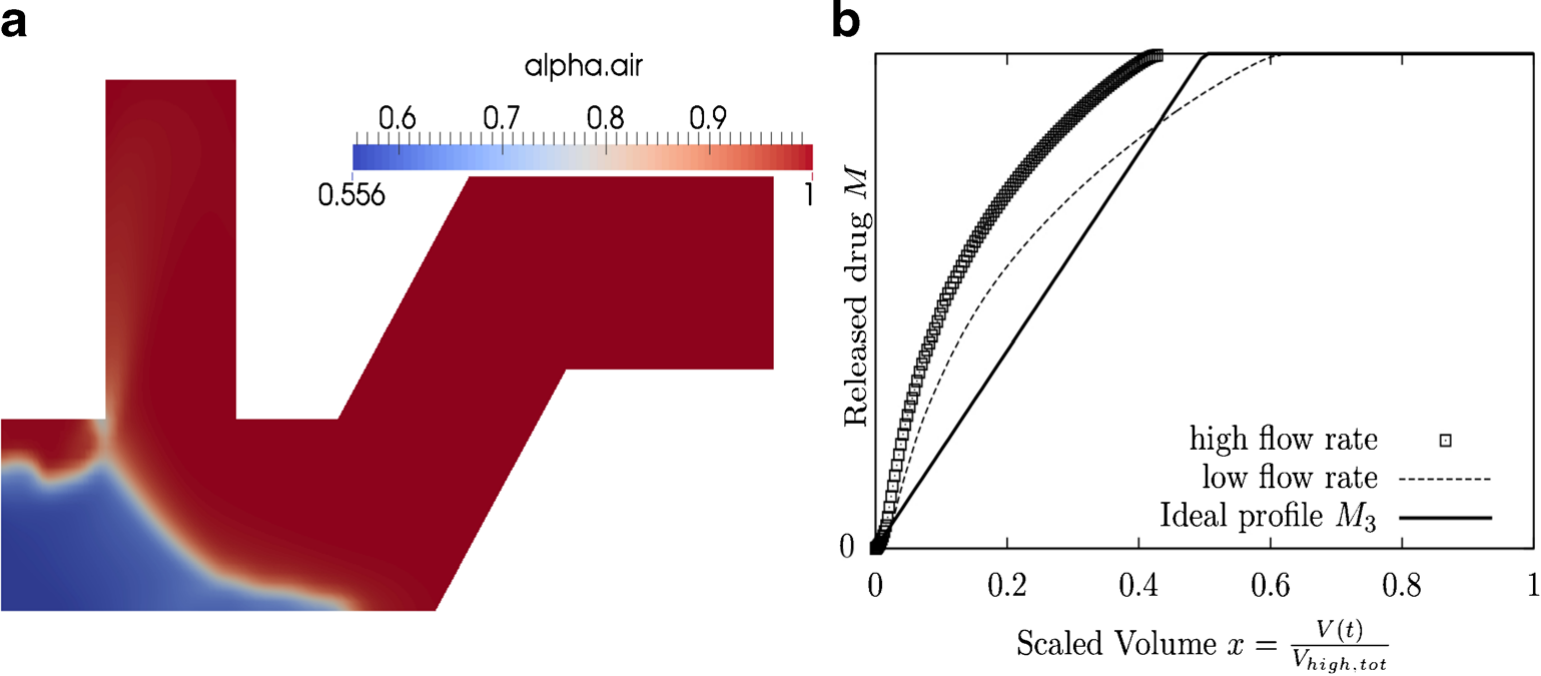
*C* = *C*_*A*_ + *C*_*B*_, *x*_0_ = 0.5, the optimal set of design variables is **x** = (0.55, 0.0, 0.79, 0.34, 0.93). (**a**)The optimal geometry and the volume fraction *α* at time *t* = 0.2s. (**b**) Scaled drug release profile for high and low flow rate.

**Fig. 10:**
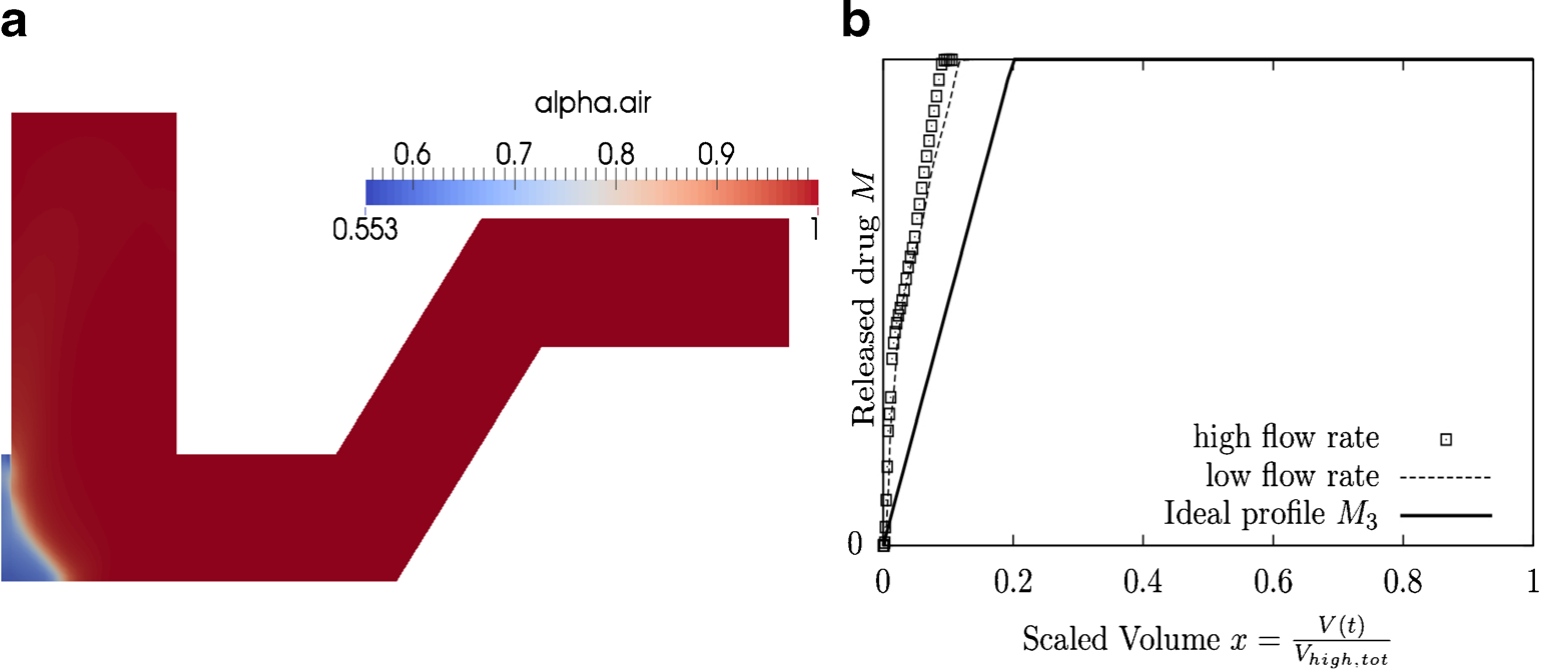
*C* = 40 *C*_*A*_ + *C*_*B*_ *x*_0_ = 0.2 and *x*_0_ = 0.5, the optimal set of design variables is **x** = (0.76, 0.29, 0.21, 0.38, 0.0). (**a**) The optimal geometry and the volume fraction *α* at time *t* = 0.2s. (**b**) Scaled drug release profile for high and low flow rates.

Optimization of the entrainment geometry of a DPI was attempted to achieve two objectives. One objective was to deliver drug to similar parts of the airways for different patients (objective A). The second objective was to deliver drug to a specific location in the airways (objective B). Optimization was conducted for four different combinations of the weightings **w** = (*w*_*A*_, *w*_*B*_), of the cost functions (*C*_*A*_ and *C*_*B*_), and the delivery duration, *x*_0_ (see Figs. 8, 9 and 10). In general, the optimized geometry depends on the weightings of the cost function **w** and the duration ${x}_0$. Figs. 8, 9 and 10 show optimization results for different values of **w** and *x*_0_.

Figure 8 shows the optimized geometry (a) and the corresponding scaled drug release profiles (b) when the cost functions were equally weighted **w** = (1, 1), *i.e. C*_*tot*_ = *C*_*A*_ + *C*_*B*_, and an early bolus delivery (*x*_0_ = 0.2) was assumed. The ‘high flow rate’ profile corresponds to the drug release profile for patient 1 with average inhalation flow, *Q*_1_ (*t*). The ‘low flow rate’ profile corresponds to the drug release profile for patient 2, with lower inhalation flow ${Q}_2(t)$. Similarly, Fig. 9 shows the optimized geometry (a) and the corresponding scaled drug release profiles (b) for the same weighting, but a more continuous drug delivery (${x}_0=0.5$). Comparing the optimized geometries for these two cases suggests that a continuous drug delivery is achieved by increasing the length of the compartment end e. Drug that is placed within the compartment end is slowly entrained by a shearing action during inhalation.

When the *C*_*A*_ and *C*_*B*_ weightings of **w** = (40,1) were chosen (*i.e*. emphasis was put on objective A to achieve maximum similarity of drug release in the same inhaled volume elements for patients with different inhalation manoeuvres), approximately the same optimized geometry was discovered, independent of the timing value ${x}_0$ that was chosen. Figure 10 shows the optimized geometry and the corresponding scaled drug release profiles when weightings of **w** = (40,1) and a timing value of *x*_0_ = 0.2 was chosen. However, it was found that very similar results were discovered for a timing value of *x*_0_ = 0.5 and thus only one figure of this type is shown. The scaled drug release profiles indicate that good agreement of release profiles of different patients was achievable, but optimization objective B, *i.e*. achieving a target release profile, was effectively ignored due to the low relative weighting. In order to achieve more continuous drug delivery higher variations in the delivery for different patients have to be accepted for this type of geometry (as shown in Fig. 9b).

For the example geometry chosen in this study, no device was found that effectively delayed the drug delivery in the scaled representation. Therefore, only a bolus-delivery at the beginning of the inhalation cycle or a continuous delivery from the beginning of the inhalation cycle are possible. Developing a passive DPI geometry that allows a delay in timing of the drug delivery will be the subject of future research.

## Conclusions

In this work, a computational method has been developed and applied for *in silico* optimization of a simple DPI geometry. Particularly, two cost functions that quantify performance of DPIs and a dynamic CFD boundary condition were developed. We have shown that CFD design optimization can be applied to DPI design. Validation is an important issue when considering numerical simulations. Due to the lack of experimental results, validation was only possible to a limited extent in this study. Nevertheless, the computational approach reported was shown to predict the drug entrainment behaviour; mechanistically, qualitatively, and with respect to the time-profile from the limited set of reported experimental observations. Multiphase CFD modelling is a challenging problem and the accuracy of predictions is limited by the quality of the model and the computational power available. The limitations of the current EE approach were that cohesive interactions and de-agglomeration of particles were ignored. In addition, only a mono-dispersed drug powder was considered in this study. However, the computational efficiency of the EE approach reported in this study shows excellent promise for modelling entrainment compared to previously reported EL approaches.

## Abbreviations

BC: Boundary Condition
CFD: Computational Fluid Dynamics
COPD: Chronic Obstructive Pulmonary Disease
DPI: Dry Powder Inhaler
EE: Eulerian-Eulerian
EL: Eulerian–Lagrangian
KTGF: Kinetic Theory for Granular Flow
PIF: Peak Inhalation Flow
